# Methodology for Controlling the Technological Process of Executing Floors Made of Cement-Based Materials

**DOI:** 10.3390/ma13040948

**Published:** 2020-02-20

**Authors:** Łukasz Sadowski, Anna Hoła, Jerzy Hoła

**Affiliations:** Department of Building Engineering, Wroclaw University of Science and Technology, Wroclaw, Wybrzeże Wyspiańskiego 27, 50-370 Wroclaw, Poland; anna.hola@pwr.edu.pl (A.H.); jerzy.hola@pwr.edu.pl (J.H.)

**Keywords:** methodology, floors, cement-based materials, casting, forming, non-destructive and semi-destructive tests

## Abstract

The article presents original complex methodology for the effective control of the entire process of executing floors made of cement-based materials. This methodology has been lacking in literature so far. The methodology was developed on the basis of many years of the authors’ experience, which was acquired when diagnosing the technical condition of such floors. The methodology was preceded by a synthetic summary of the most important technological and technical requirements for floors made of cement-based materials. It was also enriched with a discussion of the problem documented by sample research results showing the state that may be the result of disregarding and not performing the necessary control activities.

## 1. Introduction

**Figure 1:**
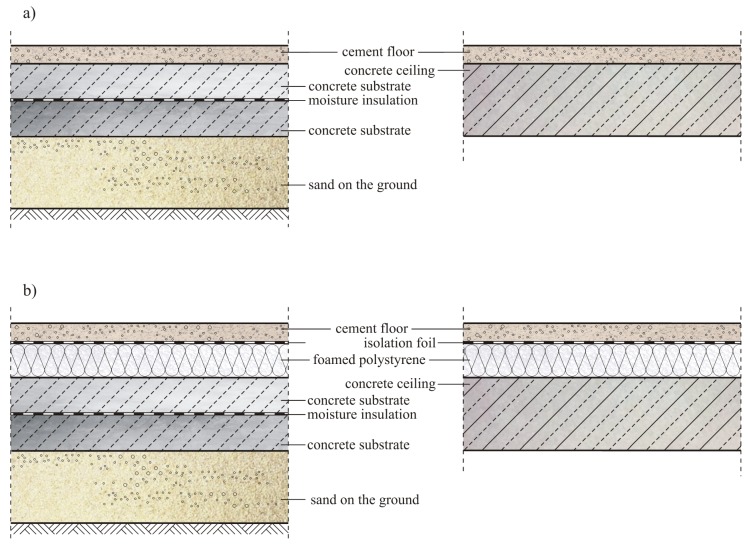
Applied variants of various floor systems made on: (**a**) a concrete substrate or a reinforced concrete ceiling: (**b**) a foamed polystyrene laid on a concrete substrate or reinforced concrete ceiling.

Currently, floors are commonly made of cement-based materials. They are used in civil engineering for various purposes, e.g., as the top layer of the floor in residential and industrial buildings, in public buildings, and in multi-car garages [1]. Floors made of cement-based materials can also be a substrate for the finishing layer of other materials, such as epoxy resin coatings, ceramic tiles, parquet, etc. [2,3,4]. The substrate for floors is usually concrete or a reinforced concrete ceiling, but can also often be foamed polystyrene, which is a layer of thermal or sound insulation, which was presented in Figure 1.

In order to make floors made of cement-based materials without faults, it is necessary to comply with the technological and technical requirements that are contained in the standards and set by the designer [5,6,7,8,9,10]. However, this is not the only condition that needs to be met. It is first necessary to ensure by the technical supervision that the conditions necessary to make a decision about the commencement of flooring are met, and then to monitor the entire implementation process on an ongoing basis. In the final phase, strength requirements should be checked before the floor is commissioned. It should be stated that in the case of strength parameters, not only the flexural and compressive strength should be monitored. It should also test the compressive strength along the thickness of the floor. The non-destructive ultrasonic method with the use of exponential heads is irreplaceable for this purpose. Ultrasounds are still very popular in civil engineering (for example for damage detection, as presented in [11]). The problem of lower compressive strength in the upper zone of floors, which is related to the horizontal direction of their concreting, is described, among others, in [12,13]. The subsurface tensile strength and abrasion resistance should also be controlled, which depends on the compressive strength in the upper zone of the floor. This upper zone of the floor is, after all, subjected to direct operational effects, and therefore maintaining an adequate compressive strength and subsurface tensile strength in this particular zone is very important for durability and safety of use [14,15,16,17]. This is very important when the floor is the final finishing layer. If the floor is laid directly on a concrete substrate or a reinforced concrete ceiling, the pull-off adhesion between the floor and the substrate also requires control, which is due to the fact that the durability of the exploited floor is dependent on this parameter [18,19,20].

In order for the entire flooring process to be correct, its control should be effective. Comprehensive control can guarantee this. This control should be complex and methodological. However, such a methodology is missing in the literature. According to the authors, the consequence of this can be seen in the very frequent cases of cement floors being executed badly, regardless of whether they are made of ready-mix cement mortars or prepared directly at the construction site [21,22,23,24]. In construction practice, there are known cases of removing defective floors and then re-executing them again [25,26,27,28,29].

Considering the above, the main goal assumed in this article is to develop—based on many years of experience acquired by the authors when testing the technical condition of floors, the quality of which has been questioned by users—an original and complex methodology for the effective control of the entire process of executing floors made of cement-based materials. The developed methodology is preceded in the article by a list of more important requirements for such floors. These are not only the result of a failure to comply with the relevant requirements, but above all the incorrect control of this technological process, which can be seen to be unmethodical, ineffective and not random. The article has been enriched with a discussion comparing and contrasting the current state and standards of quality control of floor performance with comprehensive control of the developed methodology. Its purpose is to show that the practical application of the proposed methodology will effectively eliminate many implementation problems and thus improve the quality of floors made of cement-based materials.

## 2. A Synthetic Summary of the Major Requirements for Floors Made of Cement-Based Materials

In the case of floors made of cement-based materials, they can be made of ready-mix cement mortars, or prepared directly at the construction site. Usually, fine aggregate with a grain size of up to 2 mm (e.g., quartz sand) is used to make them. In order to improve their workability, the admixtures in the form of plasticizers are used. The use of plasticizers depends on whether the mixture is laid by hand or by pumping. Table 1 presents a synthetic summary of the major technological and technical requirements for the execution of floors made of cement-based materials. These requirements are the same, regardless of whether the floors are made of ready-mix cement mortars or prepared directly on site.

## 3. Methodology of Controlling the Technological Process of Executing Floors made of Cement-Based Materials

**Figure 2:**
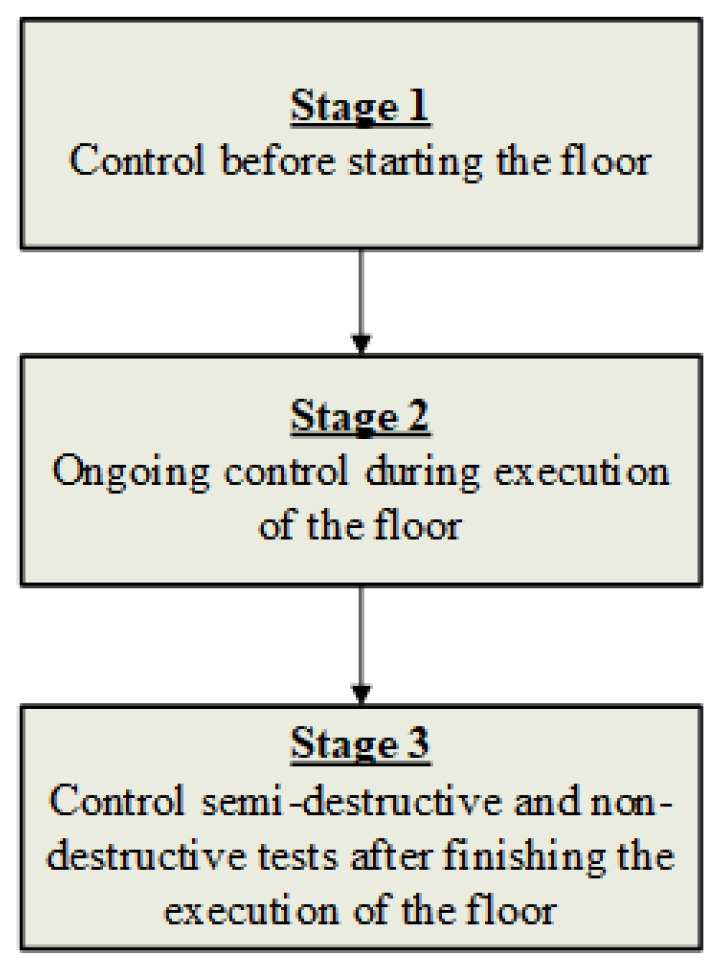
General diagram illustrating the developed methodology.

**Figure 3:**
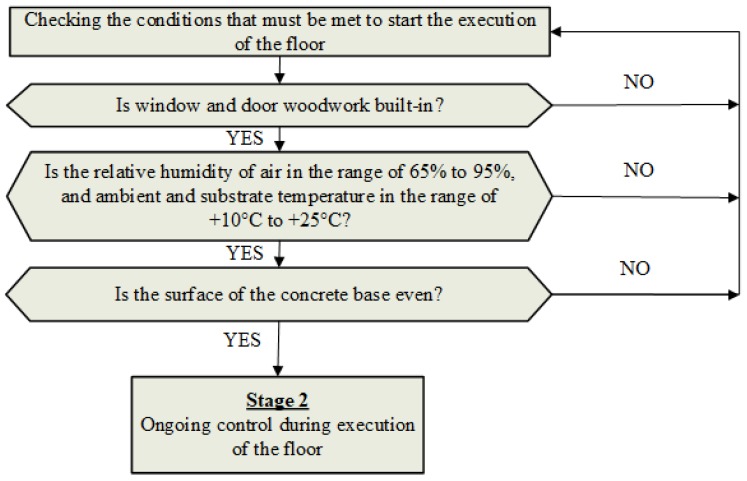
Methodology of controlling the technological process of executing floors made of cement-based materials—Stage 1 (control before starting the floor).

**Figure 4:**
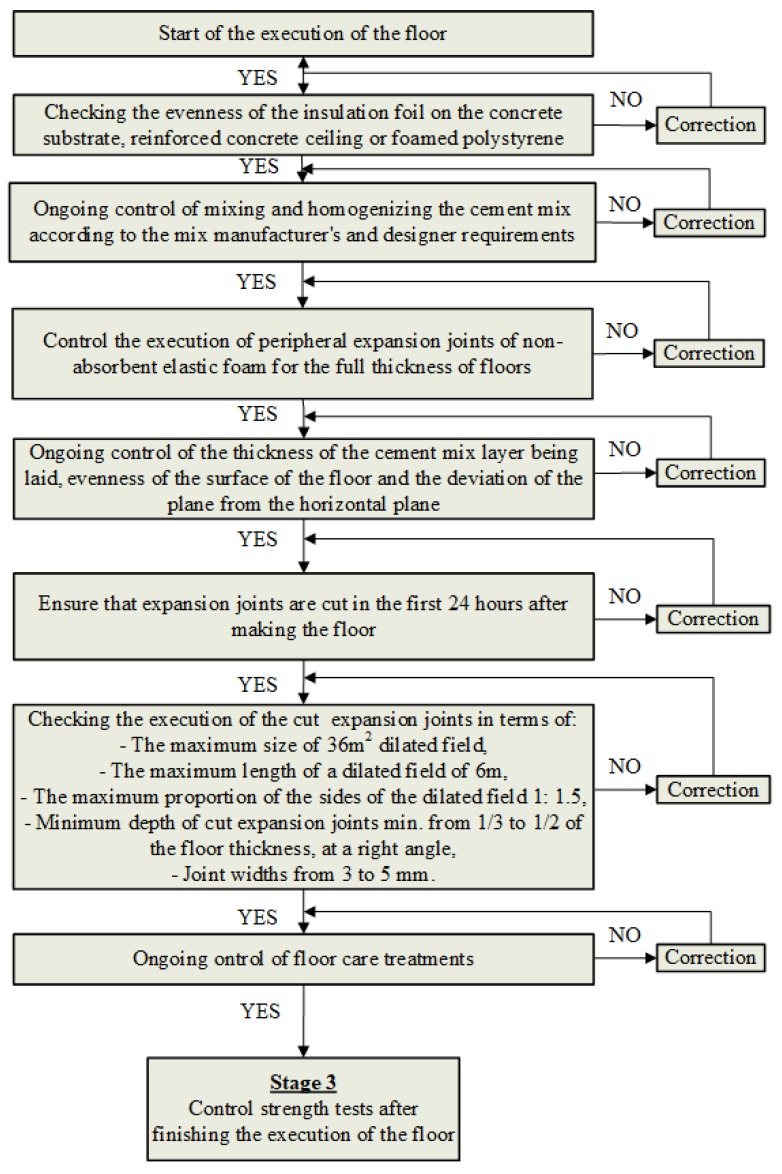
Methodology of controlling the technological process of executing floors made of cement-based materials—Stage 2 (ongoing control during execution of the floor).

**Figure 5:**
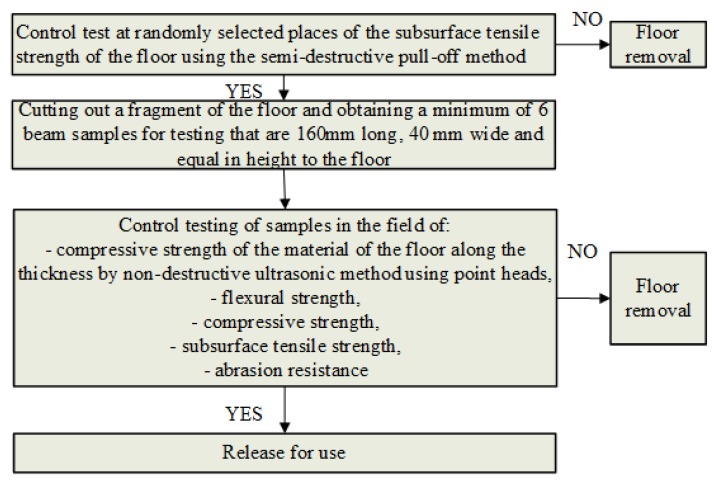
Methodology of controlling the technological process of executing floors made of cement-based materials—Stage 3 (control strength tests after finishing the execution of the floor).

The developed methodology for controlling the technological process of executing floors made of cement-based materials is described below and graphically presented in Figure 2, Figure 3, Figure 4 and Figure 5. Figure 2 presents the general, and Figure 3, Figure 4 and Figure 5 the detailed scheme of this methodology.

In Stage 1, which was presented in Figure 3, the conditions that need to be met must be checked in order to make the decision to start executing the floor. 

Stage 1 begins from checking whether the window and door joinery has been built into the building. After ensuring this condition, the relative humidity of the air in the building and the air and substrate temperature on which the floor will be laid are in the required ranges [7]. The next step is to check the evenness of the upper surface of the concrete base. Unevenness of the upper surface of the concrete base is permissible, but the upper and lower deviations need to be maintained according to [30,31,32,33,34,35]. If these conditions are met, insulating foil can be laid on the substrate. The 2nd stage then begins, in which the ongoing process of floor control takes place (Figure 4).

As it can be seen from Figure 4 the first step of Stage 2 is to check the evenness of the insulation foil on the surface of the concrete substrate, reinforced concrete ceiling or foamed polystyrene. In this step, it should be borne in mind that corrugation of the insulation foil laid under the floor is not allowed. The next step is to control the mixing and homogenization of the cement mix according to the mix manufacturer’s requirements. When laying the cement mix, the thickness of the mortar layer and the evenness of the top surface of the floor and its deviation from the horizontal plane should be checked. Peripheral expansion joints are then checked, which should be made of non-absorbent flexible foam, usually with a minimum thickness of 7 mm for the full thickness of the floors. Unevenness of the upper floor surface is only permissible if the upper and lower deviations are maintained, according to [30,31,32,33,34,35]. The next step is to make sure that expansion joints are cut in the first 24 h after executing the floor. Ongoing floor care, consisting of moisturizing it, should then be carried out. Then proceed to the control strength tests after the floor (stage 3).

**Figure 6:**
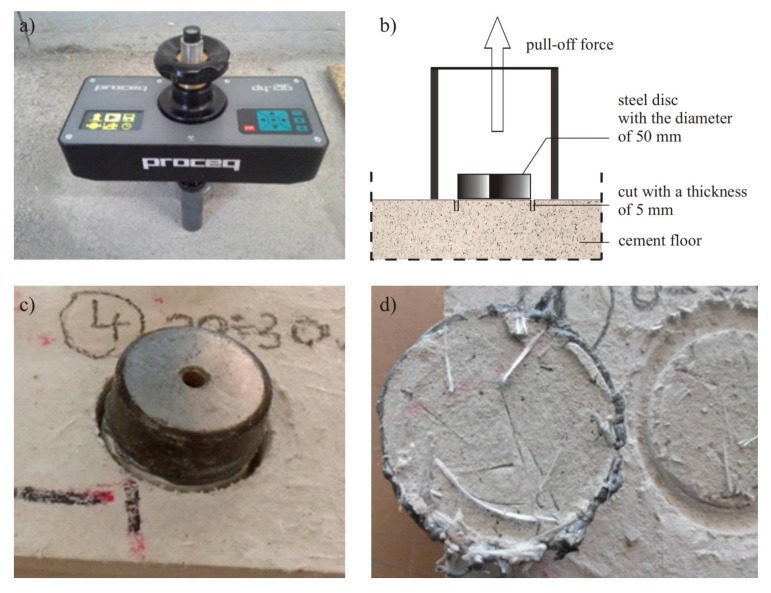
Control of the subsurface tensile strength of the floor using the pull-off method: (**a**) pull-off apparatus; (**b**) scheme of the method; (**c**) view of the glued steel disc; (**d**) view of the floor surface after the pull-off tests.

Stage 3 consists of carrying out control strength tests of the floor, without which there should be no final acceptance and commissioning (Figure 5). This stage requires a slightly broader and more detailed explanation. As illustrated in Figure 5, the first step in Stage 3 is to check the subsurface tensile strength of the floor using the semi-destructive pull-off method in randomly made places, 28 days after concreting, and in accordance with EN 1542 [36]. The pull-off tests should begin with selecting representative measuring places and preparing the surface. Then, in these places, an incision should be made in the floor at least 15 mm deep where a steel measuring disc with a diameter of 50 mm is then attached with glue. This disc should then be pulled-off from the floor (Figure 6) and the value of the subsurface tensile strength should be determined in accordance with EN 1542 [36].

**Figure 7:**
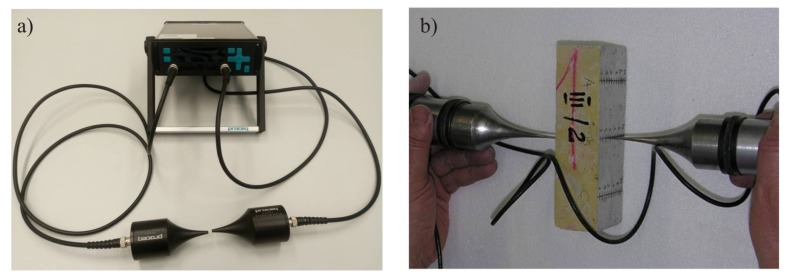
Example view: (**a**) head and probe for ultrasonic testing; (**b**) floor sample tested using the ultrasonic method.

A fragment of the floor, with such a size that at least six beam samples with a length of 160 mm, a width of 40 mm and a height equal to the floor thickness can be obtained, should then be cut off in order to check both the compressive strength of the cement mortar along the thickness and the flexural and compressive strength of the floor. The test samples should first be subjected to ultrasonic testing along the direction of concreting the floor. For ultrasonic testing, it is proposed to use an ultrasonic probe that has special exponential heads with a frequency of 40 kHz and point contact with the tested surface. According to [37] this frequency is optimum for testing cement floor samples with a dimension of 40 × 40 × 160 mm^3^. The measuring points should be applied to the lateral surfaces of the samples at a spacing of 5 mm in three rows (Figure 7).

After performing the ultrasonic tests, the lower zone of the beam samples should be cut so that their thickness is 40 mm, and then subjected to flexural and compressive strength tests. On the basis of the conducted ultrasonic tests, a correlation relationship (or possibly a hypothetical relationship from the literature) between the velocity of the longitudinal ultrasonic wave and the compressive strength of cement mortar should be developed for the tested floors. This relationship will be used to identify the course of compressive strength along the thickness of the tested floor. The compressive strength in the upper zone should not be less than 10% when compared to the strength in the middle zone. However, the flexural and compressive strength should not be less than the values for the floor that were specified by the designer. After meeting the above strength conditions, the floor can be approved for use. In the event of non-compliance with strength conditions, the floor, after consulting the designer, should be either allowed to be used, or removed.

## 4. Discussion of the Problem

As already emphasized in the introduction, the literature lacks a comprehensive methodology for controlling the process of making cement floors, which step by step would enforce subsequent control activities. This section only draws attention to those control activities, which, if neglected or omitted, result in serious flooring problems that significantly affect their final quality.

It should be started that the importance of control measures to be taken before flooring process is being started. These activities, included in Stage 1 of the developed methodology, are key to achieving a good final result.

So, currently the important problem is that it is allowed to make floors in buildings without built-in window and door joinery. This promotes the formation of drafts and intense sunlight drying of freshly laid cement mortar [38]. Due consideration is not given to the thermal and humidity conditions that should be provided in the facility, namely the required air and substrate temperature in the range from +10 to +25 °C and relative humidity in the range from 65% to 95%. Cement mortar built into the floor is not cared for by moistening it with water. These are the main reasons for the low surface compressive strength of the upper floor zone and large differences in this strength along the thickness, which is very often demonstrated on the basis of samples taken from the floors tested by the ultrasonic method. An example illustrating the above is Figure 5, which presents examples of the authors’ results of the course of the longitudinal velocity of the ultrasonic wave *c*_l_ along the thickness *h* of the cement mortar, and the course of compressive strength *f*_m_ of the cement mortar along the thickness *h* of the cement floor determined on this basis. These tests were performed by a non-destructive ultrasonic method with exponential heads with a frequency of 40 kHz.

**Figure 8:**
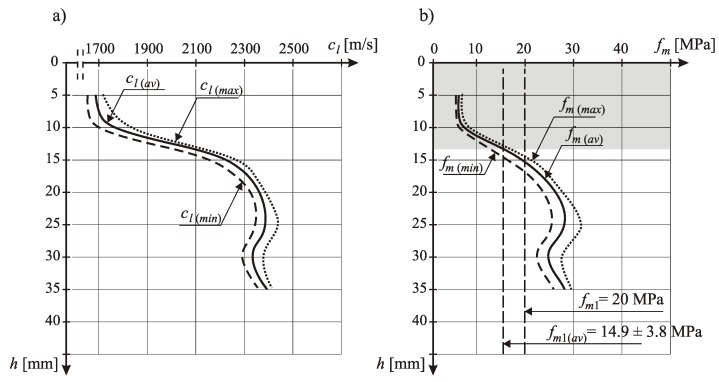
An example of the course: (**a**) the longitudinal velocity of the ultrasonic wave *c*_l_ along the thickness *h* and (**b**) the compressive strength *f*_m_ of the cement mortar along thickness *h.*

Figure 8b indicates the mean compressive strength *f*_m1_ of 20 MPa, which is required by the designer and declared by the cement mortar manufacturer, and also the average strength *f*_m_ obtained from the tests and shown with a vertical line. Figure 8 shows that within the subsurface zone of the floor up to a depth of about 15 mm from the upper floor surface, the compressive strength *f*_m_ was much lower than the strength *f*_m1_ required by the designer and declared by the manufacturer, which disqualified this floor. The occurrence of such large differences in strength along the floor thickness could not be seen in aggregate segregation, which is known and described in the literature [39,40,41,42,43,44,45].

An example is also Table 2, which shows how significantly the strength parameters of floors might differ, for which the control activities preceding its execution were neglected in relation to the requirements contained in Table 1.

**Figure 9:**
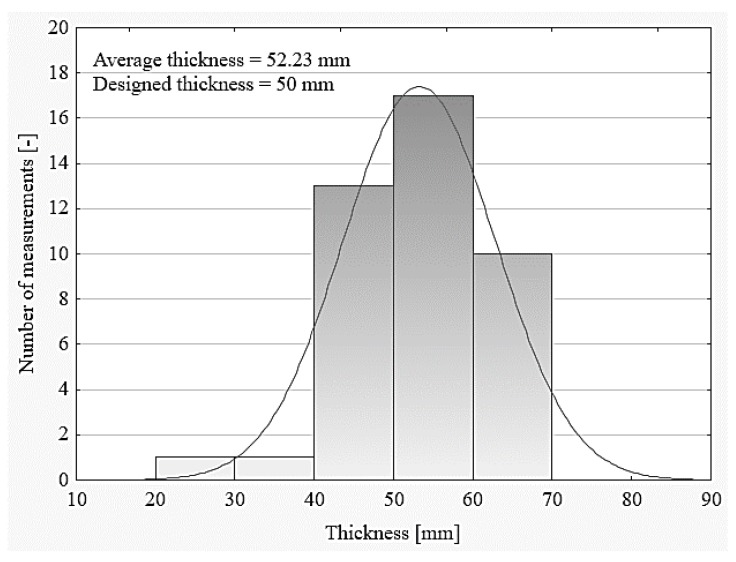
The thickness of the floors in comparison with the designed thickness (based on the data presented in [25]).

The evenness of the upper surface of the concrete base was not checked, including the occurrence of so-called local “humps”, especially interfering with the leveling of the polystyrene arrangement, which, combined with the lack of ongoing control of the thickness of the mixture being laid, resulted in obtaining a floor thickness that was not in accordance with the design. Numerous studies of the authors show that the thickness of floors is either smaller or larger in relation to the design, which is illustrated in Figure 9. Too low a thickness promotes cracking of the floor during use, whereas too large a thickness limits the floor’s load-bearing capacity.

**Figure 10:**
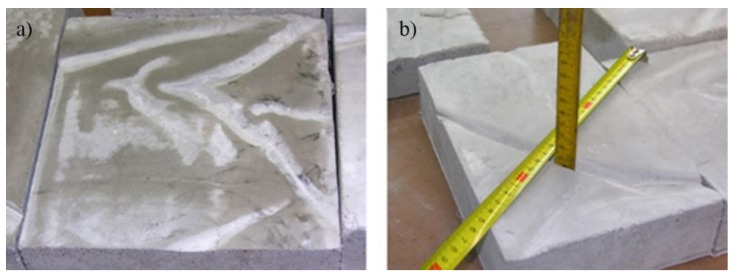
Example view: (**a**) the bottom surface of the floor, with grooved recesses constituting the “imprint” of the corrugated foil and (**b**) measuring the depth of the grooved cavities.

The problem of controlling the evenness of the insulation foil laying on the surface of a concrete substrate or polystyrene was underestimated. As a result of the corrugation of insulation foil laid under the floor on a foamed polystyrene surface, there were irregular grooved recesses on the bottom surface of the floor. As shown in Figure 10, these grooves locally reduced the thickness of the floor by a few, or even several millimeters, which promoted cracking of the floor during use.

**Figure 11:**
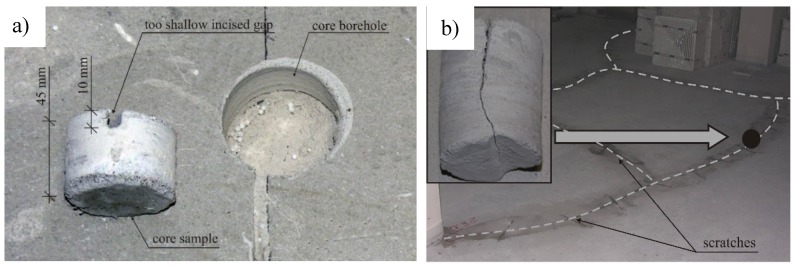
An exemplary view: (**a**) of a core borehole made in the floor, and also a core sample showing an expansion joint that is cut too shallow and (**b**) cracks on the surface of floors, along with an approximation of the cut core sample documenting the depth of one of the cracks.

In the developed methodology for controlling the process of making cement floors, in Stage 2, very much attention was paid to checking the expansion joints. Lack of such control, or sporadic controls, contribute to the incorrect execution of expansion joints, or their lack and consequent cracking of floors (for example Figure 11). Incorrect circumferential separating of floors from load-bearing walls results in a lack of continuity of expansion joints, floors being too small in width, joints being too shallow or the filling of joints with a material other than the non-absorbent flexible foam used for this purpose, e.g., multilayer cardboard.

Whereas a common disadvantage are expansion joints that are incorrectly cut, or cut at the wrong time, in cement floors. Such errors cause the dimensions of the dilated fields to be too large, the cuts to be too shallow, or the gaps to be cut too late (Figure 11). The developed methodology clearly indicates the necessary scope of control, which should exclude the problem of incorrectly made expansion joints.

In the methodology developed to control the process of making cement floors, in Stage 3, a lot of attention was paid to control strength tests after flooring. Currently, the compressive and flexural strength is being tested. At present, subsurface tensile strength, abrasion resistance and compressive strength of a cement mortar along the thickness are very rarely tested. Lack of these tests results in less durability or loosening of the applied overlay.

## 5. Conclusions

The article presents the original and effective methodology of controlling the technological process of executing floors made of cement-based materials, which there is a lack of in the literature. This methodology was developed for the entire process of the cement floor technology, which is also missing in the literature. The methodology also indicated the need to use necessary semi-destructive and non-destructive methods for such control. 

The methodology was developed on the basis of many years of experience of the authors, which was acquired during the examination of the technical condition of floors in various construction objects. This methodology has three control stages. Both the individual stages and the methodology as a whole indicate or even force, step by step, consecutive control activities to be carried out during flooring. In the discussion of the problem, attention was drawn to those selected control activities, which are currently being neglected and which result in serious implementation problems. This was documented by sample research results, clearly showing the negative effects of such omissions on the quality of the floors made.

The use in construction practice of the proposed comprehensive methodology for controlling the process of flooring made of cement-based materials should have a positive effect on the current state. It should contribute to the effective elimination of many serious implementation problems and, as a consequence, significantly improve the quality and durability of floors.

## Figures and Tables

**Table 1:** A synthetic summary of the major technological and technical requirements for the execution of floors made of cement-based materials.

Important Technological and Technical Requirements	
Floor thickness	- Compliant with the design, usually 40–60 mm.
Beginning of the execution of the floor	- After building windows and doors in the building, - In the absence of so-called drafts that overdry the cement-based material, - Without the possibility of intense sunlight or heating of the cement-based material, - At ambient and substrate temperatures between +10 and +25 °C, - At a relative humidity of 65%–95%.
Preparation of the mixture	Mechanical mixing: - without homogenizing the mixture, - with homogenizing the mixture according to standards in the mix manufacturer’s requirements.
Unevenness of the surface of a concrete substrate or reinforced concrete ceiling	- It is required not to exceed the upper and lower deviations, recorded in [30,31,32,33,34,35].
Corrugation of insulation foil laid under the floor	- It is not allowed.
The deviation of the surface from the horizontal plane	- It is required not to exceed the upper and lower deviations, recorded in [30,31,32,33,34,35].
Execution of peripheral expansion joints	- For full thickness of floors, made of non-absorbent elastic foam, - Minimum joint width 7 mm.
Execution of cut expansion joints	- Maximum field size should be 36 m^2^, - Maximum length of dilated field should be 6 m, - The maximum proportions of the sides of the dilated field 1:1.5, - The minimum depth of cut expansion joints from 1/3 to 1/2 of the floor thickness, at right angles, - Joint widths from 3 to 5 mm.
Deadline for making cut-in expansion joints	- In the first 24 h after execution of the floor.
Compressive strength	- According to the project; usually at least 20 MPa.
Flexural strength	- According to the project; usually at least 5 MPa.
Subsurface tensile strength	- According to the project; usually at least 1.5 MPa [15].
Abrasion resistance	- According to the project, usually a maximum of 22 cm^3^
Cracks	- They are not allowed [7].
Unevenness of the surface of a floor	- It is required not to exceed the upper and lower deviations, recorded in [30,31,32,33,34,35].

**Table 2:** Sample results of strength tests carried out for one of the cement floors tested in accordance with [46] (own study based on the data provided [25])**.**

Summary of Average Strength Values	
Subsurface tensile strength *f*_h_ (MPa): - Obtained on the basis of research - Required according to Table 1	0.55 ± 0.21 1.5
Compressive strength (MPa): - Obtained on the basis of research - Required according to Table 1	15.1 ± 4.5 20.0
Flexural strength (MPa): - Obtained on the basis of research - Required according to Table 1	2.3 ± 1.0 5.0
